# Potentially non-beneficial interventions in the last 100 days of life of patients with cancer: A population-based retrospective cohort study.

**DOI:** 10.23889/ijpds.v7i3.1795

**Published:** 2022-08-25

**Authors:** Colleen Webber, Abe Hafid, Anastasia Gayowsky, Michelle Howard, Peter Tanuseputro, Aaron Jones, Mary Scott, Amy Hsu, James Downar, Doug Manuel, Katrin Conen, Sarina Isenberg

**Affiliations:** 1 Ottawa Hospital Research Institute; 2 McMaster University; 3 ICES; 4 Bruyere Research Institute; 5 University of Ottawa

## Objectives

The objective of this study was to describe the receipt of potentially non-beneficial interventions in the last 100 days of life of cancer patients and to examine variations in these interventions according to patient characteristics and cancer site.

## Approach

We conducted a population-based retrospective cohort study of all adults age 18+ who died of cancer in Ontario, Canada between January 1, 2013 and December 31, 2017 using linked administrative health data held at ICES. Potentially non-beneficial interventions were captured via hospital discharge records and included chemotherapy, major surgery, intensive care unit admission, cardiopulmonary resuscitation, defibrillation, dialysis, percutaneous coronary intervention, mechanical ventilation, feeding tube placement, blood transfusion and bronchoscopy. We used bivariate analyses and multivariable Poisson regression to examine associations between the receipt of interventions and decedent age, sex, rurality, area-level income, and cancer site.

## Results

Among the 125,755 decedents, the most common intervention was blood transfusion (18.1%) and major surgery (12.8%); 23.8% received no interventions, while 14% of decedents received 3+ interventions. Lower intervention rates were observed in older patients (adjusted rate ratio (RR) 0.46, 95% confidence interval (CI) 0.44-0.49 for age 95+ vs. 19-44), females (RR 0.93, 95% CI 0.92-0.94), and individuals living in higher income areas (RR 0.96, 95% CI 0.95-0.98 for highest vs. lowest income quintile). Higher intervention rates were observed in rural patients (RR 1.13, 95% CI 1.11-1.14). Patients with pancreatic cancer had the highest intervention rate (RR 1.13, 95% CI 1.10-1.16), while breast cancer patients had the lowest intervention rate (RR 0.86, 95% CI 0.84-0.89) compared to colorectal cancer patients.

## Conclusion

Potentially non-beneficial interventions were common in the last 100 days of life of patients with cancer. Variations in interventions across patient demographics and cancer site may reflect differences in healthcare access, end-of-life care preferences, patients’ prognostic awareness, and disease factors.

